# A neglected opportunity for China’s tobacco control? Shift in smoking behavior during and after wives’ pregnancy

**DOI:** 10.1186/s12971-016-0105-8

**Published:** 2016-12-09

**Authors:** Hao Yin, Xiao Chen, Pinpin Zheng, Michelle Kegler, Qinfeng Shen, Biao Xu

**Affiliations:** 1 School of Public Health, Fudan University, Shanghai, China; 2 Key Laboratory of Public Health Safety (Fudan University), Ministry of Education, Shanghai, China; 3 University of Missouri, Kansas City, Kansas City, MO USA; 4 Health Communication Institute, Fudan University, Shanghai, China; 5 Department of Behavioral Sciences and Health Education, Rollins School of Public Health, Emory University, Atlanta, GA USA; 6 Department of Public Health Science, Karolinska Institutet, Stockholm, Sweden

**Keywords:** Smoking cessation, Behavioral medicine, Pregnancy, China

## Abstract

**Background:**

Although observational data suggest that men’s attempts and behavior at quitting smoking are often stimulated during their spouses’ pregnancy, few studies have systematically examined this phenomenon.

**Methods:**

This was a cross-sectional study which examined Chinese men’s smoking behaviors during and after their wives’ pregnancy. Women who visited community health centers for routine immunization of their children were approached. Information was mainly collected on men’s tobacco use before, during and after pregnancy in July to August 2011. Individual and socio-environmental factors were examined by non-conditional logistical regression analysis to find potential reasons behind men’s quitting during pregnancy and maintained this change till the post-partum period.

**Results:**

Totally 765 of 811 eligible women (94.3%) completed the interview. Prior to pregnancy, 42.9% of husbands smoked; this decreased to 36.34% during pregnancy, a reduction of 6.53%. Although the rate increased to a higher level (43.79%) after delivery, positive changes in men’s smoking behavior were detected. One-third (29.88%) reduced the daily number of cigarettes smoked, and nearly half (45.12%) relocated themselves to smoke when their pregnant wives were nearby. Noticeably, those who quit were most likely occasional smokers (Odds Ratio(OR) = 4.83, 95%CI [2.22, 10.48]), smoking less than ten years (OR = 2.80, 95%CI [1.19, 6.58]), not smoking at home (OR = 4.48, 95%CI [1.94, 10.39]), not smoking for social use (OR = 4.05, 95%CI [1.74, 9.41]), under lower financial pressure after the birth of child (OR = 5.28, 95%CI [2.14, 13.02]) and influenced by family members (OR = 2.82, 95%CI [1.25, 6.38]). However, only 22% of spontaneous cessation was maintained postpartum. Most relapses occurred within 6 months after delivery.

**Conclusions:**

Pregnancy offers an opportunity to decrease smoking amongst Chinese males. Intervention programs involving expectant fathers may be effective to further reduce prevalence of smoking among men in China.

## Background

Tobacco use has been recognized as a major cause of premature death for more than 50 years. Each year, over 5 million deaths are attributed to tobacco use worldwide and over 1 million occur in China [1]. The number will double by 2030 unless current trends are curbed through effective intervention strategies [2].

The benefits of quitting smoking increase over time, with significant reductions in the risk of numerous adverse health outcomes [3]. In 2008, The World Health Organization (WHO) highlighted the MPOWER strategy (in brief: monitor use, protect people, offer help, warn dangers, enforce bans and raise taxes) aiming to end the global tobacco epidemic [4]. Cessation assistance is one of the key strategies for bringing the anti-tobacco war to the ultimate stage. However, quitting is not yet normative in China [5]. According to the Global Adult Tobacco Survey (GATS) China, 2010, the quitting ratio in ever daily smokers was 12.8% — the second lowest amongst all GATS countries [6]. In 2010, China’s ever smokers had a 33.1% relapse rate, similar to that in 2002 and 1996. Three out of four smokers (75.6%) had no plan to quit smoking and the main reason for thinking about quitting smoking was concern for personal health (55.0%) [7]. These findings suggest that, in the past decade, little improvement has been realized since the majority of smokers would not quit until they were faced with a health problem. Thus, there is an urgent need for better outcomes in China’s tobacco control.

Experts suggest that pregnancy offers a teachable moment in which male partners may be particularly receptive to cessation messages [8]. A moderate decrease (3–5%) in smoking prevalence amongst male smokers during their partners’ pregnancy was reported in Norway [9]. Several studies conducted in Asia-Pacific regions also found a significant decrease [5]. However, similar decreases were not observed in other European countries and the North American region, indicating the potential influence of cultural differences which calls for further investigation [10, 11].

Spontaneous quitting has been observed in young men during their wives’ pregnancy in the United States [12]. Several studies have acknowledged that pregnancy can motivate smoking husbands to quit [13–15]. A systematic review showed that, regardless of the various quitting ratios across studies, men’s attempts at quitting smoking were often stimulated during their spouses’ pregnancy [16]. In addition to cessation, improvements have been observed in fathers’ smoking patterns. Many smoked outside the room to avoid exposing the mother and the infant to second-hand smoke. Pregnancy and delivery also offered an opportunity for men to strengthen social responsibility for their family’s health status. Each year, China witnesses more than 16 million newborns [17] suggesting that the benefits could be considerable if practical action was taken to encourage expectant fathers to quit smoking during this critical period.

Nonetheless, very limited information is available in China on this topic. One study in Guangzhou documented that some husbands did spontaneously quit smoking when their wives were pregnant [18]. Yet in this research, quitting was defined as not smoking in the past 7 days, which excluded possibility of relapse. Our previous study on exposures to Environment Tobacco Smoke (ETS) in China’s new mothers revealed that 10.7% of smoking husbands reported quitting smoking during their wives’ pregnancy [19]. These findings were comparable to some intervention effects, but no further information was collected to study the reasons behind the cessation success. Neither were data collected to assess whether cessation was sustained after delivery. Thus, the objective of the current study was to understand the extent to which men quit smoking during and after their wife’s pregnancy; to determine the associated factors; and to assess whether cessation was sustained after delivery in terms of cumulative prevalence of relapse.

## Method

### Participants

Five community health centers in two districts of Shanghai were purposively selected to represent both urban and suburban populations. During July to August 2011, all mothers with children aged 3–18 months who came to the community health center for childhood vaccinations under the national immunization program were asked by the doctor or nurse on site to participate in this study. It was assumed that 38% of the male smokers might quit smoking or reduce tobacco consumption, and the no response rate of the participating women was 10%, thus, the sample size was estimated as 718 (652 + 66). According to the annual report of Shanghai Municipal Center for Disease Prevention and Control, the national immunization program had a nearly full coverage (100%) in Shanghai suggesting good representativeness [20]. Women who had been living with a husband/partner in Shanghai for at least one year before the last pregnancy, and also living together during pregnancy and at least three months after were eligible for participation regardless of having a valid Shanghai residence identity.

### Procedure and survey content

Eligible mothers were asked to complete a self-administrated anonymous questionnaire after their child’s vaccinations were completed. Trained medical students were on site for assistance. The questionnaire included not only questions regarding socio-demographics; women’s own smoking status and that of their husbands and extended family members; ETS exposure at home; requests made to husband/partner to stop smoking in their presence; knowledge and attitude about smoking and second hand smoke; but also men’s tobacco smoking and quitting during pregnancy; counselling receiving. Counselling refers to education on harms of tobacco towards the pregnant women and husbands which was but not limited to counselling from doctors, midwives and other health professionals.

For those with a husband who smoked, additional information regarding the husband’s quitting behavior during and after the pregnancy was collected. This information included, but was not limited to alterations in numbers and locations of smoking, and factors the mother believed were related to these alterations. Additionally, Women who reported that their husband quit smoking during their pregnancy were asked to report whether a relapse had happened after delivery and the duration between delivery and relapse. In the context of Chinese culture, men living with their female partners in this study were called “husband” regardless of marital status.

We hypothesized that both individual and socio-environmental factors could contribute to expectant fathers’ quitting smoking during pregnancy. Therefore, we listed individual reasons such as age, education, income and current or ever smoker; and socio-environmental reasons such as influences from family members, working environment, social context and living pressure.

### Definition of measures

Ever smokers were persons who smoked at least 100 cigarettes in their lifetime. Current smokers were ever smokers who were smoking at the time of the survey. Smoking status was classified as daily smokers or occasional smokers. Daily smokers were further classified as light, moderate or heavy smokers for less than 10, 10–20 and more than 20 cigarettes smoked per day respectively, in accordance with the definition used in GATS China 2010 and 2002 National Surveys [6, 21]. Occasional smokers were defined as persons who were currently smoking some days during a week. Quitting smoking was defined as smokers who had not smoked for at least three months.

Environmental factors were assessed with the following items: “My husband quit smoking because he cannot stand the price of cigarettes”, “My husband quit smoking because we discourage his smoking including persuading him not to smoke, setting non-smoking rules at home or asking him to smoke in other places away from me”; “My husband cannot quit smoking because he needs to use cigarettes as a means for social interactions”, “My husband cannot quit smoking because he needs to use cigarettes as a relief for the enhanced financial pressure of being a father” (since the newborn would force him to be financially supportive to the family as a man in the context of China). The five available responses were “do not agree, slightly agree, somewhat agree, mostly agree, completely agree”. We dichotomized these responses into do not agree, slightly agree, somewhat agree in one group, and mostly agree, completely agree in the other group.

### Statistical analyses

Descriptive statistics were used to describe participants’ demographic characteristics, knowledge and beliefs about smoking, their partners’ smoking prevalence before, during and after pregnancy, and the percentage that had quit smoking. Chi-square tests were used for univariate comparisons and non-conditional logistical regression analysis was applied to identify demographic and socio-economic factors associated with quitting and relapse. Crude and adjusted odds ratios (OR) were calculated with 95% confidence interval (95%CI). All the statistical analyses were performed with SPSS Version 19.0 (SPSS, Chicago, IL, USA).

## Results

All 832 post-natal mothers who attended the clinic for child vaccination were approached during the study period. Twenty-one of the total 832 women did not meet the eligibility criteria (two were under the age of 18 and nineteen had resided in Shanghai for less than one year). Forty-six females refused to participate for reasons including insufficient time available, concerns about information disclosure and lack of interest. Consequently, 765 women completed the questionnaire, with a response rate of 94.3%.

The average age of the women surveyed was 28.6 (SD = 3.1) years. Over half (56.4%) had a college degree. Compared with nonsmoking husbands, husbands who smoked were less likely to have an education beyond high school (*P* < 0.0001) or to hold a valid Shanghai residence (*P* < 0.0001), and more likely to have monthly income less than 5000 CNY (*P* < 0.0001, equivalent of 750 USD). No statistically significant differences were observed between male smokers and nonsmokers regarding age, study site or frequency of receiving counseling on quitting smoking during wives’ pregnancy (Table 1).

**Table 1:** Characteristics of men’s smoking status reported by their wives

Characteristics	Nonsmokers (*n* = 403)		Smokers (*n* = 362)		*P* Value
	No.	%	No.	%	
Age (years)					
< 30	111	27.5	104	28.7	0.326
30–35	230	57.1	199	54.9	
35+	62	15.4	59	16.2	
Education					
Secondary School or Less	55	13.6	95	26.2	<0.0001
High School Graduate	65	16.1	101	27.9	
College Graduate	160	39.7	112	30.9	
Above college	123	30.5	54	14.9	
Residence					
Shanghai	207	51.4	134	37.0	<0.0001
Non-Shanghai	196	48.6	228	63.0	
Study site					
District 1	171	42.4	176	48.6	0.086
District 2	232	57.6	186	51.4	
Household income/M (CNY)^ab^					
< 5000	105	26.6	140	39.5	<0.0001
5000–10000	119	30.1	121	34.2	
10000+	171	43.3	93	26.3	
Counseling received during pregnancy					
Yes	286	71.0	249	68.8	0.511
No	117	29.0	113	31.2	

^a^Household income per month, 1 USD = 6.2 CNY

^b^Missing value = 16

Three hundred and sixty-two of the 765 respondents (47.32%) reported their husbands as current or ever smokers. The prevalence of husband smoking in one year before, during and at least three months after their wives’ pregnancy were 42.87% (328/765), 36.34% (*n* = 278/765) and 43.79% (*n* = 335/765) respectively. Of the male smokers the average number of cigarettes smoked per day at these three stages was 9.16, 7.22 and 8.48 respectively. During their wives’ pregnancy, a large proportion of the 328 smoking husbands altered their smoking behaviors: 15.24% (50/328) quit smoking, 29.88% (98/328) reduced the daily number of cigarettes smoked, and 45.12% (148/328) relocated to smoke when their wives were nearby. In contrast, only 8.54% (28/328) of the smoking husbands made no behavior change, whereas 1.22% (4/328) increased the number of cigarettes smoked. According to the age of infants, husbands were classified into three group: 3–6 months, 6–12months and 12–18months. Despite initial changes, however, smoking relapse happened at 75% (12/16), 50% (7/14) and 85% (17/20) of the husbands respectively. The average proportion of quitting was 78% and most of the relapses occurred in the first six months after delivery (Table 2).

**Table 2:** The proportion of smoking relapse in men by the age of child (Months)

Time after the birth of infant (months)	Proportion of relapse by the age of child		
	3–6	6–12	12–18
0–3	11	2	5
3–6	1	3	11
6–12	-	2	0
12–18	-	-	1
None	4	7	3
Total	16	14	20
Relapse Rate	75%	50%	85%

To those whose husband were smokers, 70% of the women reported that they had received counseling and/or information during pregnancy from doctors, co-workers, mass media and Internet but no significant association was found with the husbands’ quitting (*P* = 0.24). The top three reasons that women believe in hindering husband to quit smoking were tobacco addiction (52%), not knowing how to quit (50%) and social context (46%). Another 48% agreed that smoking causes addiction and therefore medical assistance is necessary.

Multivariate analysis was conducted to understand the association between individual and environmental factors and quitting smoking (Table 3). After adjusting for potential confounders, those who quit smoking during their wives’ pregnancy were more likely those who were occasional smokers (OR = 4.83, 95%CI [2.22, 10.48]), who had smoked less than ten years (OR = 2.80, 95%CI [1.19, 6.58]), who did not smoke at home (OR = 4.48, 95%CI [1.94, 10.39]), did not use cigarettes as a social tool (OR = 4.05, 95%CI [1.74, 9.41]), who did not report an enhanced financial pressure of being a father (OR = 5.28, 95%CI [2.14, 13.02]), and who were influenced by family members (OR = 2.82, 95%CI [1.25, 6.38]).

**Table 3:** Multivariate analysis for factors associated with spouses quitting during their wives pregnancy, China (*n*=328)

Factors	Quitting smoking		Adjusted OR^a^	*P*-value
	Yes	No	(95%CI)	
Age (years)				
< 30	13	82	1.00	0.653
30–35	29	148	1.07 (0.44,2.58)	
35+	8	48	1.70 (0.51,5.63)	
Education				
< =Secondary School	6	80	1.00	0.451
High School	19	70	1.05 (0.23,4.76)	
College	17	87	1.94 (0.54,6.96)	
Above College	8	41	0.86 (0.27,2.71)	
Income (CNY)				
< 5000	20	109	1.00	0.151
5000–10000	12	97	0.40 (0.14,1.13)	
10000+	18	72	0.74 (0.27,2.55)	
Residence				
Non-Shanghai	29	176	1.00	0.033
Shanghai	21	102	2.76 (1.08,7.04)	
Study site				
District 1	30	130	1.00	0.039
District 2	20	148	2.24 (1.04,4.85)	
Smoking status before pregnancy				
Daily smoker	15	220	1.00	<0.0001
Occasional smoker	35	58	4.83 (2.22,10.48)	
Smoking years				
≥ 10	14	154	1.00	0.018
< 10	36	124	2.80 (1.19, 6.58)	
Smoking at home				
Yes	15	174	1.00	<0.0001
No	35	104	4.48 (1.94,10.39)	
Wife received education on harms of tobacco				
No	13	90	1.00	0.238
Yes	37	188	1.68 (0.71,4.01)	
Social use				
Yes	17	152	1.00	0.001
No	33	126	4.05 (1.74,9.41)	
Under financial pressure				
High	21	83	1.00	<0.0001
Low	29	195	5.28 (2.14,13.02)	
Perception of cigarette price				
Expensive	37	202	1.00	0.245
Not expensive	13	76	1.70 (0.70,4.01)	
Influenced by family members^b^				
No	18	154	1.00	0.013
Yes	32	124	2.82 (1.25,6.38)	

^a^Adjusted for age, education, income, residence and study site

^b^This includes persuading husband not to smoke, setting rules at home or asking husbands to smoke in a designated area

## Discussion

Pregnancy offers a compelling opportunity for both pregnant women and their partners to quit smoking. Capitalizing on pregnancy as a teachable moment could serve as an effective tool for China to curb the tobacco epidemic and save thousands of lives. Nonetheless, this opportunity has been overlooked at least in part due to limited research and insufficient evidence supporting this approach. It is fortunate that prevalence of female smoking is rather low in China (2.4%), in comparison to the great prevalence in the male population (52.9%) [6].

This study mainly focused on partner/husband smoking. It provided descriptive information about married male smokers’ tobacco use before, during and after their wives’ pregnancy. We found the proportion of smoking husbands that smoked post-pregnancy was high (43.79%), in comparison to other low-and-middle-income countries [22], and that smoking husbands were more likely to be middle aged, less educated and at a lower social-economic status.

Spontaneous quitting, often recognized as the Cold Turkey method, is viewed as the least effective method with a success rate of only 4–7% [23]. However, results in this study indicate that even without cessation support (that is medication, behavior intervention and cessation counseling), pregnancy substantially increased (15.24%) husbands’ smoking cessation rate. At the same time, many of the husbands decided to cut down the number of cigarettes smoked per day (29.88%) or change their smoking location (45.12%), whereas less than one out of ten sustained smoking at the same amount (8.54%) or raised the number of cigarettes smoked (1.22%). According to a report from a randomized control trial, men’s cessation rate at the 6^th^ month of pregnancy reached up to 16.5%, compared to 9.3% in a control group without any planned interventions [24]. This suggests the benefit of interventions that target male smokers during their wives’ pregnancy is considerable, especially on the basis of China’s enormous smoking population. In addition, more than two thirds of the female respondents in this study reported that they had received counseling and/or information during pregnancy from doctors, co-workers, mass media and Internet that suggested the creation of a smoke-free family at pregnancy. With convincing messages and evidence-based approaches from credible sources, pregnant women can be empowered and contribute collectively to improve husbands’ smoking behavior.

It is notable that a large proportion of male smokers choose to change their smoking location (45.12%), whereas they were unwilling to quit smoking or cut down cigarettes smoked. Second hand smoking during pregnancy is a risk factor for both the mother and the embryo. Abundant evidence indicated the correlation between nicotine exposure and multiple adverse outcomes as preterm delivery and stillbirth [25, 26]. Previous research has found that the main source of ETS exposure to women is their smoking husbands and other family members; and smoking males, especially fathers were encouraged to take precautions when a pregnant woman was nearby [19]. This slight movement of smoking outdoors rather than inside would largely reduce ETS exposure not only for the pregnant mother but also for the unborn infant [27]. Simultaneously, male smokers were encouraged to reconsider their tobacco use, concerning the health of their family members. Therefore, pregnancy offers a golden opportunity for effective interventions launched on maternal exposure to second hand smoking at pregnancy.

Perinatal abstinence from smoking is associated with both individual and environmental factors [28–30]. Our study found that individual factors like occasional smokers, and not smoking at home increased quit rates. Environmental factors like non-social use, low financial pressure from having a new baby and presence of family intervention were also significantly associated with men’s quitting. Policies like mass media campaigns and bans on smoking in public places have also influenced the climate of social smoking. On the other hand, over two third of women received education on the harms of tobacco during pregnancy, but no significant association was found in our study regarding husbands’ quitting. As far as our understanding goes, such education does not provide direct intervention to smoking husbands, rather, a reminder of harms of smoking to pregnant women with no follow up instructions or supports. Meanwhile, most wives in this study were not sure if their husbands had received any kind of quitting assistance from practitioners or any other medical professional. In fact, although evidence-based interventions have already been proved to be effective, the methods themselves haven’t reached the majority of China’s population. Most significantly, wives usually don’t recognize nicotine addiction as a latent obstacle against their husbands’ quitting. Only 48% of women agreed with the notion that smoking causes addiction and requires medical assistance to help smokers to quit.

The main limitation of this study was the reliability of the wives’ reporting on behalf of their husbands. Additionally, the convenience sampling might have influenced the representativeness of the study population, and the cross-sectional design could have incurred potential recall bias, to some extent, as women were asked to recall their partners’ smoking habit at pregnancy. It is also very likely that women did not remember the exact number of cigarettes their husbands smoked per day before, during and after their pregnancy. Therefore we asked them to answer the approximate numbers such as 5, 10, 15 or 20. It is also possible that women were not sure how much their husband smoked at work or outside the home. However, a previous study conducted in China found that wives were able to correctly report their husbands’ smoking behavior [31]. Additionally, inclusion criteria only covered those women who lived with their husbands during pregnancy, one year before and at least three months after, thus increasing the likelihood that respondents could answer the full set of smoking questions.

Last but not least, four out of five quitters relapsed after the delivery. This indicates that smoking cessation at pregnancy might not be sustainable in the long run. However, this study is limited regarding the details. No information was collected on why spontaneous quitting at pregnancy was not enough to both radically and “permanently” change husbands’ smoking behavior, since only smokers abstinence of smoking for two years could be regarded as successful quitters [32]. One possibility was that pregnancy, which lasts for about ten months, does not provide a sufficient amount of time for husband to reconstruct their smoking behavior. This study’s findings suggested that professional supports for expectant fathers who have already initiated quitting smoking are urgently needed to stimulate and maintain the quitting progress.

## Conclusion

Pregnancy is a neglected opportunity for Chinese men to quit smoking. A 15.24% quitting rate was observed in expectant fathers in this study, along with another 75% changing their smoking behavior in some way. However, cessation was followed by a 78% cumulative relapse after delivery. Tobacco control interventions during pregnancy should be tailored to meet the needs of both the husband and the wife. They should be individualized respectively for light and heavy smokers. Professional and societal supports should be offered to the new parents to sustain quitting. A tobacco-free environment could make remarkable impacts on men’s quitting smoking during their wives’ pregnancy.
